# Different Domains of the RNA Polymerase of Infectious Bursal Disease Virus Contribute to Virulence

**DOI:** 10.1371/journal.pone.0028064

**Published:** 2012-01-13

**Authors:** Cyril Le Nouën, Didier Toquin, Hermann Müller, Rüdiger Raue, Katherine M. Kean, Patrick Langlois, Martine Cherbonnel, Nicolas Eterradossi

**Affiliations:** 1 Avian and Rabbit Virology, Immunology and Parasitology Unit, OIE Reference Laboratory for Infectious Bursal Disease, French Agency for Food, Environmental and Occupational Health Safety (Anses), Ploufragan, France; 2 Institute for Virology, Faculty of Veterinary Medicine, University of Leipzig, Leipzig, Germany; 3 CNRS URA 3015, Institut Pasteur, Paris, France; 4 Virus Genetics and Biosecurity Unit, French Agency for Food, Environmental and Occupational Health Safety (ANSES), Ploufragan, France; INRA, France

## Abstract

**Background:**

Infectious bursal disease virus (IBDV) is a pathogen of worldwide significance to the poultry industry. IBDV has a bi-segmented double-stranded RNA genome. Segments A and B encode the capsid, ribonucleoprotein and non-structural proteins, or the virus polymerase (RdRp), respectively. Since the late eighties, very virulent (vv) IBDV strains have emerged in Europe inducing up to 60% mortality. Although some progress has been made in understanding the molecular biology of IBDV, the molecular basis for the pathogenicity of vvIBDV is still not fully understood.

**Methodology, Principal Findings:**

Strain 88180 belongs to a lineage of pathogenic IBDV phylogenetically related to vvIBDV. By reverse genetics, we rescued a molecular clone (mc88180), as pathogenic as its parent strain. To study the molecular basis for 88180 pathogenicity, we constructed and characterized *in vivo* reassortant or mosaic recombinant viruses derived from the 88180 and the attenuated Cu-1 IBDV strains. The reassortant virus rescued from segments A of 88180 (A88) and B of Cu-1 (BCU1) was milder than mc88180 showing that segment B is involved in 88180 pathogenicity. Next, the exchange of different regions of BCU1 with their counterparts in B88 in association with A88 did not fully restore a virulence equivalent to mc88180. This demonstrated that several regions if not the whole B88 are essential for the *in vivo* pathogenicity of 88180.

**Conclusion, Significance:**

The present results show that different domains of the RdRp, are essential for the *in vivo* pathogenicity of IBDV, independently of the replication efficiency of the mosaic viruses.

## Introduction

Infectious bursal disease virus (IBDV) is a pathogen of worldwide significance to the poultry industry. IBDV destroys the immature B lymphocytes in the bursa of Fabricius (BF) of chickens, thus causing immunosuppression, and clinical disease mainly occurs between 3 and 7 weeks of age [1]. Since the late eighties, very virulent (vv) IBDV strains have emerged in Europe inducing up to 60% mortality [2]–[4].

IBDV is a bisegmented double stranded (ds) RNA virus belonging to the family *Birnaviridae*, genus *Avibirnavirus* [5]. Genome segment A (3,2 kbp) encodes in a large open reading frame (ORF) a precursor polyprotein which is cleaved by autoproteolysis to yield ***i)*** the outer capsid, VP2, the maturation, tri dimensional structure and major immunogenic determinants of which have been described [6]–[9], ***ii)*** the virus protease, VP4 [10], [11] and ***iii)*** an internal protein, VP3, that binds to viral dsRNA to form ribonucleoprotein complexes [12]. From a small ORF preceding and partially overlapping the large ORF, segment A also encodes VP5, a non structural protein [13] possibly involved in virus release [14]–[16] and/or inhibition of apoptosis at early stages of infection [17]. Genome segment B (2,8 kbp) encodes the virus RNA dependent RNA polymerase (RdRp or VP1) [18], the polymerase activity of which has been unequivocally characterized *in vitro* [19]. The RdRp is also present in virus particles as a genome linked protein (VPg) [20]. The recently determined tri-dimensional structure of the IBDV RdRp showed that the polypeptide chain can be divided into three domains: ***i)*** an N-terminal domain (residues 1–167) ***ii)*** the central polymerase domain (residues 168–658) which contains the “Fingers”, “Palm” and “Thumb” subdomains and ***iii)*** a C-terminal domain (residues 659–878) [21], [22]. The comparison of the tri-dimensional structure and the aminopeptidic sequence of IBDV VP1 with RdRp of other RNA viruses [21]–[24], including IPNV [25], allowed to identify eight amino acids motifs supposed to be essential for the biological activity of IBDV RdRp. These motifs include the putative self-guanylylation site of IBDV RdRp in the N-terminal domain of the polymease [25], [26]. The 7 others motifs are present in the central domain of the polymerase and are suggested to be involved in the nucleotide recognition and binding (motif A, B and F), phosphoryl transfer (motif A and C), control of metal dependence (motif C), structural integrity of the palm (motif D), priming nucleotide binding and proper positioning of the “Thumb” relative to the “Palm” (motif E), and template binding (motif B, F and G) [21]–[23], [27]–[34]. Interestingly, the C motif was found to be in a permuted position in the RdRp of *Birnaviridae* as compared with other viral RdRps [21]–[24] which could be linked with an unusual Co^2+^ dependence of this polymerase [34].

Although some progress has been made in understanding the molecular biology of IBDV, the molecular basis for the pathogenicity of vvIBDV is still not fully understood. The study by reverse genetics of the genes or genomic regions of vvIBDVs involved in pathogenicity is particularly challenging because these viruses are not adapted to cell culture. Currently, the vvIBDV phenotype is still largely defined by a significantly increased mortality following challenge. Segment A-encoded proteins do play a role, as demonstrated by reverse genetics studies where a mosaic classical virus containing the VP2 of a vvIBDV strain did induce lesions of the BF. However the mosaic virus failed to induce either morbidity or mortality in young chickens, thus demonstrating that VP2 of vvIBDV was not the sole determinant for their pathogenicity. In addition, the introduction of VP4 or VP3 of a vvIBDV into the genetic background of a classical virus did not confer any pathogenicity [35]. Another study with interserotypic reassortant viruses generated by reverse genetics confirmed that segment A of serotype 1 IBDV was responsible for the bursa tropism [36].

The literature regarding the influence of segment B on IBDV phenotype reveals somewhat conflicting results. It was suggested in an early study that segment B of vvIBDV has a limited influence on pathogenicity [35]. However, other studies showed that segment B of a vvIBDV, a variant or a vaccine IBDV strain could influence the replication efficiency and modulate the bursal lesions [36]–[38]. The latter hypothesis seems consistent with the phylogeny-based assertion that both genome segments may be involved in IBDV pathogenicity [39] and with the observations that naturally occurring reassortant IBDVs with either a vvIBDV-related segment A but an unrelated segment B [40], or the converse genetic make up (classical segment A with vvIBDV-related segment B, [41], [42], exhibit a reduced or increased pathogenicity, respectively. A chrono-phylogenetic study also suggested that the worldwide expansion of vvIBDV likely started following the acquisition of a new vvIBDV-related segment B, whereas the vvIBDV-related segment A could have been introduced several years earlier [43].

In the present study, we investigated the molecular basis for the pathogenicity of IBDV strain 88180, previously characterized in our laboratory as the only known representative of a lineage of pathogenic IBDV with significant phylogenetic relationships to vvIBDV [40], [44]. We used the reverse genetics approach developed by Mundt and Vakharia [45] to construct infectious cDNA copies of the 88180 genome. These plasmids allowed the rescue of mc88180, a molecular clone with the same pathogenicity as its parental strain. In order to investigate the molecular basis of pathogenicity in mc88180, the plasmids were then used in reverse genetics to construct reassortant or intra segment B mosaic viruses derived from the mc88180 and the attenuated Cu-1 IBDV strains. Our results confirm that both genome segments are important for a full pathogenicity and further demonstrate that several (if not all) regions within segment B are also required.

## Results

### The two genome segments of 88180 are more related to their counterparts in vvIBDVs than to the attenuated Cu-1 strain

Because this study provided the first full length sequences of an IBDV isolate belonging to an original genetic lineage [44], the nt or aa sequences (for non coding [NCR] and coding regions, respectively) of the full-length segments A or B segments of the bursa derived 88180 strain were compared in detail with their counterparts in the attenuated Cu-1 strain [36] and in typical vvIBDV D6948 strain (Table 1).

**Table 1:** Location of the nucleotide (non coding regions) or amino acid (coding regions) differences in both genome segments between 88180, Cu-1 and typical vvIBDV strain D6948 (Acc No *AF240686* and *AF240687* for segments A and B, respectively).

Segment A					Segment B				
Nt or aa position	Cu-1	88180	D6948	Gene or region	Nt or aa position	Cu-1	88180	D6948	Gene or region
44	t	c		**5′NCR**	55	c	t		**5′NCR**
45		c	t		58	t	a	t	
47		t	a		59	a	g		
69	g	a			60	a	t	c	
78	t	c	t		63	g	a		
79	t	a	t		69	g	a		
86		c	t		106		g	a	
−1	-	-	M	**VP5**	4	I	V		**Domain 1 (N-ter; F1)**
−2	-	-	L		13	T	K		
−3	-	-	S		35	R	K		
−4	-	-	L		41	V	I	E	
14	K	E			55		K	R	
34	N	H	N		145	N	T		
45		G	R		146	E	*D*		
74		I	F		147	G	S	*N*	
125	S	P			219	D	N	D	
133	R	W			242	D	*E*		
222	P	Q	*A*	**pVP2**	287	T	A		
242	V	I			305	S	N	S	
253	H	Q			390	L	*M*		**Catalytic domain (P1-F2-P2)**
256	V	*I*			391	A	T	A	
257	L	M	L		393		E	*D*	
270	T	A			508	R	K		**Domain 2 (P2-T-C-ter)**
279	N	D			511	R	S		
284	T	A			546	L	P		
294		L	*I*		562	S	F	*P*	
297	S	P			576	T	A	T	
299		N	*S*		595	C	S	C	
300	E	Q	E		646	G	S		
330	R	S			687	S	*P*		
334	A	V	A		695	K	*R*		
451	I	L			756	K	R	K	
541	V	I		**VP4**	879	-	Q		
680	C	Y			2749	c	t		**3′NCR**
685		K	N		2786	t	c		
691	A	T			2827	-	c	-	
713	A	T							
715	P	S							
751	H	D							
981	L	P		**VP3**					
990	A	V	A						
1005	T	A							
3168		a	g	**3′NCR**					
3205	t	c							
3224	t	g	a						
3229	c	a							
3231	t	a							
3261	t	c	-						
3262	-	c	-						

The 88180 sequence was used as a reference and only nucleotides or amino acids differences were presented. Italicised letters indicate positions that have been reported to be typical of vvIBDV, in VP2 and VP1. For the segment A, in the left column, −1, to −4 numbers correspond to the presence of four additional amino acids in the VP5 protein of the typical vvIBDV strain D6948 (153 aa long) compare to 88180 or Cu1 strains (149 aa long). In the second large ORF partly overlapping VP5, pVP2 corresponds to aa 1 to 512, VP4 to aa 513 to 755 and VP3 to aa 756 to 1012. For the segment B (878 aa), domain 1 contains the N-terminal domain (N-ter) and Finger 1 (F1) subdomains, the catalytic domain contain the Palm 1 (P1), Finger 2 (F2) and Palm 2 (P2) sub-domains and domain 2 contains the Palm 2 (P2), Thumb sub-domains (T) and the C-terminal region (C-ter) as determined from the tridimensional structure of the IBDV polymerase recently solved [21], [22].

In segment A, the sequence of the 5′ NCR of 88180 exhibited 4 or 5 nt changes as compared with Cu-1 or D6948, respectively. The overall predicted secondary structure of the A5′NCR was more similar with that of Cu-1 and one nucleotide change found in 88180 (44C) restored a predicted stretch of paired nucleotides (nts 10 to 16, base-paired with nts 40 to 46) also predicted in D6948 (data not shown). The predicted VP5 protein of 88180 (4 and 7 aa differences with Cu-1 and D6948, respectively) was four aa shorter than in D6948 at its N-terminal extremity, as also found in Cu-1, and exhibited several aa changes in its putative cytoplasmic domain. In the predicted polyprotein, the VP2 precursor (pVP2) of 88180 differed from Cu-1 and D6948 at 13 and 6 aa positions, respectively. All but one of these changes were located in the so-called “antigenic domain” of VP2, and 3 of the differences observed with D6948 affected aa positions conserved in typical vvIBDV (A222, I294 and S299). The only other change (L451→I, observed in Cu-1) affected the pep46 peptide released upon the maturation process of VP2. The deduced VP4 protein of 88180 was much similar with VP4 of D6948 (only one aa change), but exhibited 6 aa changes as compared with Cu-1, two of these changes (aa 541 and 751) occurring in regions of the protease possibly important for the cleavage at the VP4-VP3 and pVP2-VP4 junctions, respectively. VP3 of 88180 exhibited 1 aa change as compared to its D6948 counterpart in the putative RNA-fixation site (aa 990) and 3 aa changes as compared with Cu-1. Two of the 3 latter changes (aa 981 and 990) were present in the putative RNA-fixation site and one (aa 1005) in the VP1 interaction site. Finally, nts changes occurring in the 3′NCR of segment A did not appear to cause drastic changes in the predicted secondary structure of the NCR (data not shown).

**Figure 1:**
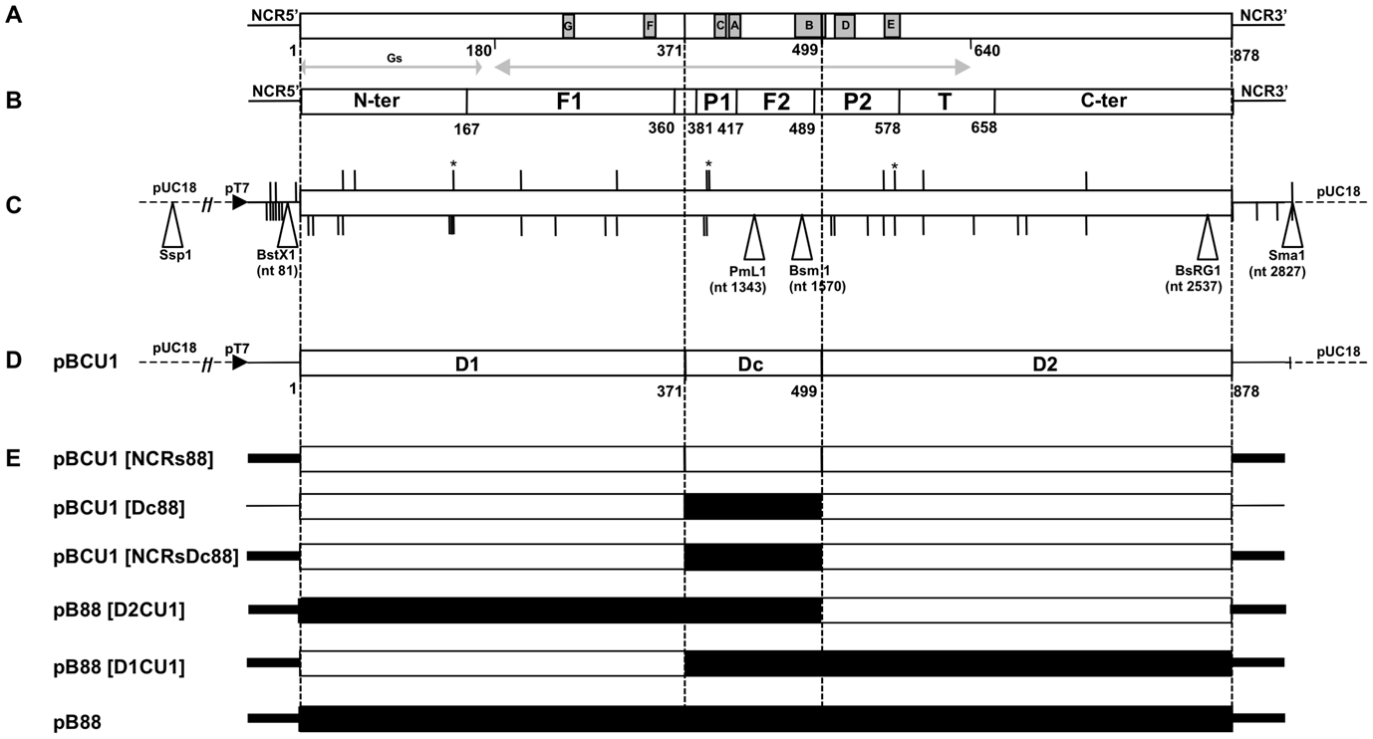
Organisation of IBDV segment B and construction of mosaic B segments derived from pB88 and pBCU1. **A**) Position of the motifs involved in the biological activity of IBDV RdRp. The first double arrow (aa 1 to 175) shows the possible region where the self-guanylylation site (Gs) of IBDV RdRp is predicted to be positioned [26]. The 7 others motifs (G, F, C, A, B, D and E) are present in the central domain of the polymerase and are suggested to be involved in the nucleotide recognition and binding (motif A, B and F), phosphoryl transfer (motif A and C), control of metal dependence (motif C), structural integrity of the palm (motif D), priming nucleotide binding and proper positioning of the “Thumb” relative to the “Palm” (motif E), and template binding (motif B, F and G) (see text for references). The second double arrow indicates the domain of VP1 interacting with VP3 [55]. Finally, aa 371 to 499 have been defined as a region with alternating β layers and **α** helixes corresponding to the Palm 1 and Finger 2 region delimiting the catalytic domain. **B**) The tri-dimensional structure of the IBDV RdRp recently solved allowed to determine that the polypeptide chain can be divided into three domains: *i)* an N-terminal domain (residues 1–167) surrounding *ii)* the central polymerase domain (residues 168–658) which contains the “fingers” (F1 and F2), “palm” (P1 and P2) and “thumb” (T) subdomains and *iii)* a C-terminal domain (residues 659–878) [21], [22]. **C**) Segment B of 88180 cloned into the pUC18 plasmid under the control of the T7 promotor (horizontal black triangle). The position of nts (NCRs) or aa (coding region) that differentiate 88180 from pBCU1 or D6948 are indicated by short black vertical bars appearing below or above the plasmid, respectively. Asterisks identify conserved aa typical of vvIBDV [39] that are modified in 88180. White vertical triangles indicate the position of unique and conserved enzymes restrictions sites used for the construction of pBCU1/pB88 mosaic plasmids. **D**) The two regions of IBDV VP1 (D1 and D2) surrounding the catalytic domain (DC) considered for the construction of mosaic segment B, together with the aa position of their boundaries. **E**) The constructed mosaic B segments. Black and opened areas represent genome regions derived from pB88 and pBCU1, respectively. See in text the method of construction.

In segment B, the 5′ NCR of 88180 exhibited 6 and 3 nt changes as compared with Cu-1 and D6948, respectively. None of these combinations of changes modified the predicted secondary structure of the NCR. Interestingly, this structure included the base-pairing of nts 53–56 with nts 62–65, hence forming a hairpin structure close to a terminal loop, and base-pairing was maintained at these positions although both nts of the 55–63 pair were modified in Cu-1 (being C-G, as compared with T-A in 88180 or D6948). This suggests that the secondary structure at this position could be important for the function of the B5′ NCR. Comparison of the VP1 aa sequence of the three viruses revealed 23 aa differences between Cu-1 and 88180, and 11 differences between 88180 and D6948. In both comparisons, most amino acid changes were quite evenly distributed along the ORF, however both pairs of viruses exhibited aa differences located close to the catalytic domain of VP1 (changes aa positions 390–391 and 391–393 in Cu-1 and D6948, respectively). Out of the 8 aa positions that had been previously reported to be unique and common to all vvIBDV [39], five were conserved in 88180 (D146, E242, M390, P687, R695), but three were modified in this virus N147→S, D393→E and P562→F) (see asteriks in Figure 1 C). Finally, the 3′NCR of B88 had the same nt sequence as its D6948 counterpart (and hence the same predicted secondary structure), whereas the predicted structure of the B3′ NCR in Cu-1 exhibited some differences due to the C2786→T nucleotide change.

The nt sequences of the A and B genome segments of 88180 were finally compared, using phylogenetic methods, with their counterparts in a panel of vvIBDV and classical strains retrieved from databanks [46]. This phylogenetic study confirmed that the segments A and B of 88180 are both phylogenetically related to, but nevertheless significantly different from, vvIBDV (data not shown).

### mc88180 exhibits the same phenotype as its parental 88180 strain

The antigenicity of mc88180 was first compared with that of the 88180 virus. Both viruses exhibited the same antigenic profile as previously reported (with a lack of binding of monoclonal antibodies [Mabs] 3 and 4 and a strong reactivity of all other Mabs) [44], thus showing that the aa change introduced in mc88180 at VP2 aa position 281 does not alter the virus reactivity with the studied Mabs (data not shown).

**Figure 2:**
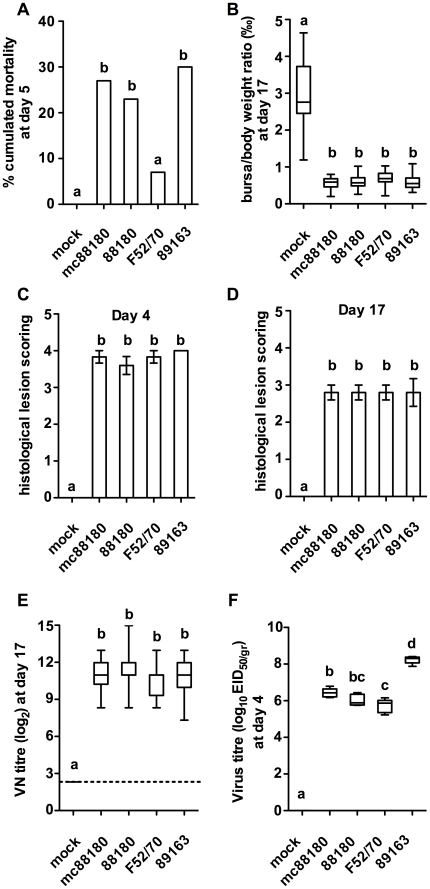
Compared pathogenicity of recombinant or wild type IBDV strains mc88180, 88180, F52/70 and 89163 in six-week old SPF chickens (experiment *i*). On the first day of the experiment, SPF chickens were housed in five groups of 30 chickens of comparable sex and weight. On the same day, each bird was inoculated by the intranasal route with a standardized dose of the relevant virus (10^5^ EID_50_ by bird). A group was kept as a mock-inoculated control receiving PBS only. See in Table S3 the summarized protocol of the animal experiment. **A**) % cumulated mortality at 5 days post infection, **B**) BF to body weights ratio (‰) at day 17 (n = 21 to 25 chickens depending on the group). The box plots show the median (horizontal line) flanked by the 2^nd^ and 3^rd^ quartile. The outer bars show the range of values. **C**) and **D**) Histological lesion scoring at day 4 and 17, respectively (n = 5 to 6 chickens per group). The outer bars show the range of values. **E**) VN titer at day 17 (in log_2_, n = 21 to 25 chickens depending on the group). **F**) virus titer at day 4 post infection (in log_10_ EID_50/gr_, n = 5 chickens per group). Treatments sharing the same lowercase letter do not differ significantly according to the kruskal-Wallis test (BF to body weights ratio, lesion score, VN titer and virus titer) or according to the Chi-squared test (% mortality), at the p≤0.05 confidence level.

The pathogenicity of mc88180 was then compared under standardized experimental conditions with that of IBDV strains 88180, F52/70 and 89163, the two latter strains being used as controls selected for their typical classical or vv IBDV phenotypes, inducing 5–20% and 20–60% mortality under the studied experimental conditions, respectively (Figure 2). Neither clinical signs nor mortality were observed in the mock-inoculated control group which remained seronegative throughout the experiment. In the groups receiving the challenge viruses, typical signs of acute IBD (diarrhoea, prostration, ruffled feathers) were observed from 3 to 7 days post inoculation (DPI) and mortality occurred from 3 to 5 DPI (Figure 2 A). The Chi-square test showed that mortalities induced by the mc88180 (27%), 88180 (23%) and 89163 (30%) viruses did not differ significantly but were significantly higher than that induced by the F52/70 challenge (7%). The only other difference between the four inoculated viruses was in the titer of virus recovered from the bursae sampled at 4 DPI (Figure 2F), as 89163 resulted in 10^8.2^ EID_50_/g whereas the three other viruses yielded comparable and significantly lower virus titers (10^6.4^, 10^6.0^, and 10^5.7^ EID_50_/g for the mc88180, 88180 and F52/70 viruses, respectively). None of the other observed criteria (mean bursa-to-body weight [b/B] ratio, mean bursal lesion score [BLS] at 4 and 17 DPI, and geometric mean VN titer at 17 DPI, Figure 2B, C, D and E) did reveal any significant difference between the four challenged groups. Altogether, these results suggested that mc88180 indeed induced the same acute pathogenicity as the parental 88180 strain.

### Both segments of mc88180 are essential for a full pathogenicity

**Figure 3:**
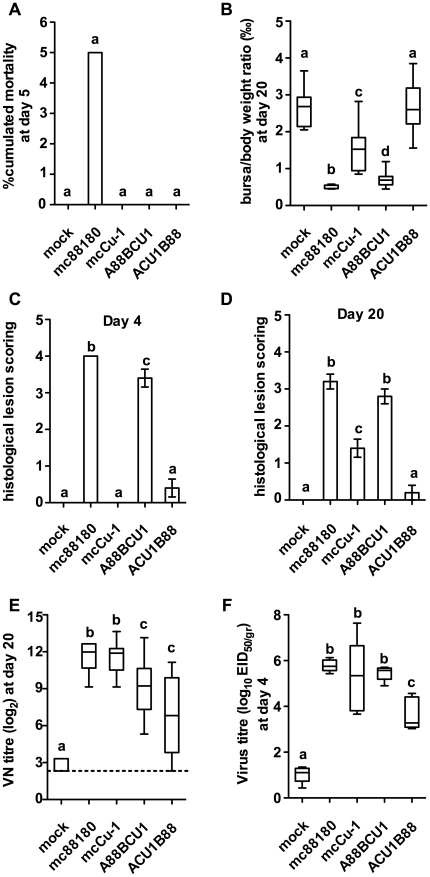
Compared pathogenicity of mc88180, mcCu-1, A88BCU1 and ACU1B88 in seven-week old SPF chickens (experiment *ii*). On the first day of the experiment, SPF chickens were housed in five groups of 20 chickens of comparable sex and weight. On the same day, each bird was inoculated by the intranasal route with a standardized dose of the relevant virus (10^5^ EID_50_ by bird, except for the ACU1 B88 virus which was inoculated with 10^3.6^ EID_50_ by bird). A group was kept as a mock-inoculated control receiving PBS only. See in Table S3 the summarized protocol of the animal experiment. **A**) % cumulated mortality at 5 days post infection, **B**) BF to body weights ratio (‰) at day 20 (n = 7 to 10 chickens depending on the group). The box plots show the median (horizontal line) flanked by the 2^nd^ and 3^rd^ quartile. The outer bars show the range of values. **C**) and **D**) Histological lesion scoring at day 4 and 20, respectively (n = 5 chickens per group). The outer bars show the range of values. **E**) VN titer at day 20 (in log_2_, n = 10 chickens per group). **F**) Virus titer at day 4 (in log_10_ EID_50/gr_, n = 5 chickens per group). Treatments sharing the same lowercase letter do not differ significantly according to the kruskal-Wallis test (BF to body weights ratio, lesion score, VN titer and virus titer) or according to the Chi-squared test (% mortality), at the p≤0.05 confidence level.

In order to investigate which segment of mc88180 was involved in pathogenicity we produced by reverse genetics the mcCu-1 virus and the two reassortant viruses derived from mc88180 and mcCu-1, namely A88 BCU1 and ACU1 B88 (Suplementary Table S1). All viruses were inoculated under standardized experimental conditions to 7-week-old SPF chickens. Results of this experiment are presented in Figure 3.

Neither signs nor mortality were observed in the mock-inoculated control group. Morbidity was observed from 3 to 5 DPI: mc88180 induced 100% morbidity, A88BCU1 40% (p≤0.001 as compared with mc88180), ACU1B88 5% and mcCu-1 0%. The two latter groups did not differ significantly with respect to morbidity, but differed significantly (p≤0.05) from both mc88180 and A88BCU1 (data not shown). Only mc88180 induced mortality, although at a lower rate than in the previous experiment (5%) (Figure 3A).

Analysis of the other criteria (see Figure 3 B, C, D, E and F) confirmed that the mock-inoculated control group remained uninfected throughout the experiment, and that the molecularly cloned viruses performed consistently with the phenotype of their parent viruses. Indeed, mcCu-1 replicated well in the bursa (genome amounts equivalent to 10^5.3±1.6^ EID_50_/g detected in qRT-PCR, Figure 3F), induced significant but limited bursal lesions (mean b/B ratio and BLS at 20 DPI = 1.5±0.6‰ and 1.4±0.5, respectively, Figures 3B and 3C) and proved highly immunogenic (mean VN titer at 20 DPI = 11.5±1.3 log_2_, Figure 3E), in agreement with Zierenberg et al. (2004) [36]. As also expected, mc88180 replicated in the bursa to a similar extent (10^5.8±0.3^ EID_50_/g detected in qRT-PCR) and proved both highly pathogenic (inducing the smallest mean b/B ratio at 20 DPI, 0.5±0.1‰, and the highest BLS at both 4 and 20 DPI, 4.0±0.0 and 3.2±0.4, respectively) and immunogenic (mean VN titer 11.6±1.3 log_2_). The two reassortant viruses exhibited markedly different properties. On the one hand, ACU1B88 replicated to a significantly lower extent in the bursa (10^3.7±0.7^ EID_50_/g detected in qRT-PCR, p = 0.013), it hardly induced any bursal lesion (no significant difference in b/B ratio or in BLS with uninfected controls, irrespective of the sampling date) and proved poorly immunogenic (highly heterogeneous VN titers at 20 DPI, ranging <3.3 to 11.1). These results suggested an infectivity even lower than that of mcCu-1 which induced significantly more bursal atrophy, histological lesions and antibodies at 20 DPI (see above, p≤0.05). On the other hand, A88BCU1 replicated as well as the mc viruses (10^5.5±0.3^ EID_50_/g detected in qRT-PCR) and induced marked bursal atrophy (mean b/B ratio at 20 DPI = 0.7+0.2‰), severe histological lesions (mean BLS at 4 and 20 DPI = 3.4±0.5 and 2.8±0.4, respectively) and high levels of antibodies (geometric mean VN titer = 9.2±2.3 log_2_).

Taken together, our results showed the low compatibility of the segment A of CU1 with the segment B of 88180 and also confirmed the paramount role of segment A in the pathogenicity. However, all criteria but the virus titer indicated also a slight but significant (p≤0.05) reduction of the pathogenicity of A88BCU1 as compared with mc88180 (Figure 3), in agreement with the reduced morbidity observed at 4 DPI. These results showed that segment B of 88180 is also involved in the pathogenicity of mc88180.

### The association of several regions of segment B of 88180 is required for pathogenicity

**Figure 4:**
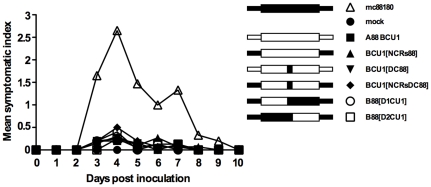
Mean individual symptomatic index between 1 to 10 day post inoculation in chickens inoculated with mosaic recombinant IBDV strains. The symptomatic index was calculated according a scale ranging from 0 to 3 with, with 0 meaning “lack of signs”, 1 meaning “typical IBD signs (ruffled feathers) conspicuous in quiet bird only, the bird stimulated by a sudden change in environment (light, noise, or vicinity of experiment observer) appears normal, motility is not reduced”; 2 meaning “typical IBD signs conspicuous even when bird is stimulated, dehydration is apparent, motility may be slightly reduced” and 3 standing for “typical severe IBD signs with prostration or death”. mc88180 induced significantly more morbidity than all other viruses from 3 to 9 DPI (p≤0.0001 to p≤0.003), whereas no significant difference in the severity of clinical signs was observed between the groups receiving the other viruses or mock-inoculated., A simplified representation of the chimeric segments B (based on the ones described in Figure 1) used in association with the segment A of 88180 to generate the indicated mosaic recombinant viruses is shown.

**Figure 5:**
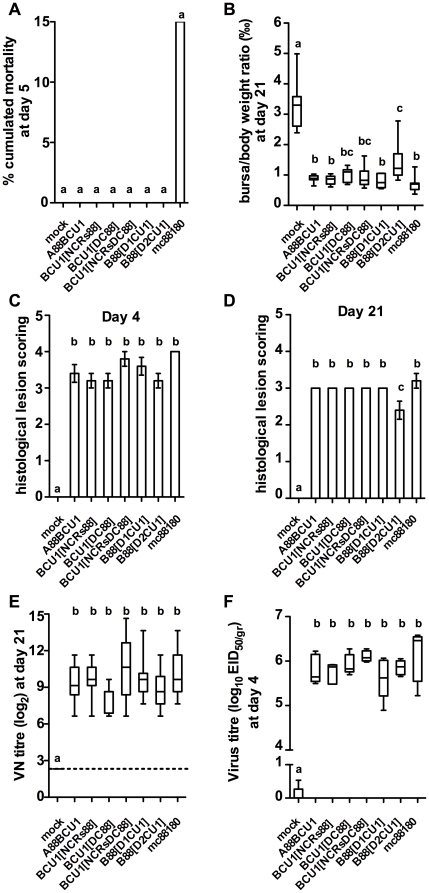
Compared pathogenicity of mc88180, A88BCU1 and five recombinant viruses with mosaic segment B in five-week-old SPF chickens (experiment *iii*). See in Table S1 and Figure 1 the genetic make up of the viruses with mosaic segment B and in Table S3 the summarized protocol of the animal experiment. On the first day of the experiment, SPF chickens were housed in eight groups of 20 chickens of comparable sex and weight. On the same day, each bird was inoculated by the intranasal route with a standardized dose of the relevant virus (10^5^ EID_50_ by bird). A group was kept as a mock-inoculated control receiving PBS only. **A**) % cumulated mortality at 5 days post infection, **B**) BF to body weights ratio (‰) at day 21 (n = 10 chickens per group). The box plots show the median (horizontal line) flanked by the 2^nd^ and 3^rd^ quartile. The outer bars show the range of values. **C**) and **D**) Histological lesion scoring at day 4 and 21, respectively (n = 5 chickens per group). The outer bars show the range of values. **E**) VN titer at day 21 (in log_2_, n = 8 to 10 chickens depending on the group). **F**) Virus titer at day 4 (in log_10_ EID_50/gr_, n = 5 chickens per group). Treatments sharing the same lowercase letter do not differ significantly according to the kruskal-Wallis test (BF to body weights ratio, lesion score, VN titer and virus titer) or according to the Chi-squared test (% mortality), at the p≤0.05 confidence level.

The critical role of segment A and domains of this segment involved in vvIBDV pathogenicity was previously investigated [35]. However, the domains of the IBDV polymerase involved in virulence remain unknown. To investigate which regions of the segment B of A88BCU1 could be responsible for this virus reduced pathogenicity, we tried to restore it in recombinant mosaic IBDVs in which the A88 background was kept but different regions of segment B of CU1 were replaced with their counterparts in B88 (Figure 1). Five mosaic B segments were constructed (Figure 1E) and the resulting viruses were rescued (Table S1) and investigated *in vivo* for their pathogenicity as before. As in the previous experiments, the mock-inoculated group kept uninfected throughout the experiment. Calculation of a newly designed mean symptomatic index (see materials and methods) showed that mc88180 (maximum mean symptomatic index = 2.6 at 4 DPI) induced significantly more morbidity than all other viruses (mean symptomatic index = 0.2 to 0.5 at 4 DPI, p≤0.0001) (Figure 4). This difference kept significant from 3 to 9 DPI (p≤0.0001 to p≤0.003), whereas no significant difference in the severity of clinical signs was observed between the groups receiving the other viruses or mock-inoculated. Once again, only mc88180 induced mortality (15%, no significant difference with 0% observed in other groups, Figure 5A).

At 4 DPI, the seven inoculated groups did not differ significantly by their mean BLS (ranging 3.2±0.4 to 4.0±0.0, Figure 5C) and by the amounts of virus genome detected in BF (equivalent to virus titers ranging 10^5.6±0.5^ to 10^6.1±0.6^ log_10_ EID_50_/g, Figure 5F), thus showing that all the viruses with a mosaic segment B replicated well in the bursa. At 21 DPI, no significant difference in the antibody levels were observed in the birds inoculated with the seven viruses (Figure 5E). The chickens inoculated with B88[D2CU1] seemed to experience a quicker recovery than the other groups, as illustrated by significantly higher b/B ratio (1.4±0.6‰, Figure 5B) and significantly lower BLS (2.4±0.5, Figure 5D) at 21 DPI than in the other inoculated groups, which did not differ significantly one to another (mean b/B ratios ranging 0.7±0.5 to 1.0±0.2‰ and mean BLS ranging 3.0±0.0 to 3.2±0.4, p≤0.05).

## Discussion

As an attempt to investigate the molecular basis for IBDV pathogenicity, mc88180, the molecular clone of a vvIBDV-related pathogenic IBDV strain [40], [44] was rescued from plasmid copies of both genome segments.

The mc88180 virus was shown to exhibit the same phenotype as its parental 88180 strain, as demonstrated by a similar antigenic reactivity and ability to induce morbidity and mortality. However, some variation in the mortality induced by mc88180 was observed in our three animal experiments, as the mc88180-induced mortality ranged 5 to 27% (no statistically significant difference according to the Chi-squared test). Although mortality in the first experiment (27%) did not differ significantly from that induced by the 89163 vvIBDV control virus (30%), the percent mortality observed in subsequent experiments was lower than would have been expected in our experimental model for a typical vvIBDV [44], [47], [48]. Whether some genetic specificities of 88180 might be associated with a reduced pathogenicity as compared with vvIBDV will be discussed below. The variation in successive experiments cannot be explained by the source of the SPF chickens (AFSSA-Ploufragan), nor by their age (as they had been selected around the maximum susceptibility age of 6 weeks), nor by the virus titers in the mc88180 inoculum (which was the same virus suspension). However, the sequencing of the virus pools recovered from the BF of chickens succombing to mc88180-infection in each experiment showed that in experiment ***i)*** only, a mixed virus population was detected. Indeed, at aa position 281, the virus pool contained the Glutamic acid used as a genetic tag for mc88180 as well as the Glycin corresponding to the aa found in wild-type 88180. This result shows that mc88180 partly reverted to the wild-type sequence in experiment ***i)***, which could explain the highest pathogenicity of mc88180 observed in this specific experiment. Further studies would be required to assess precisely the role of aa 281 in 88180 pathogenicity. Whatever this may be, the intensity of the clinical signs induced by mc88180 in the successive experiments was enough to investigate the role of the segments A and B in the pathogenicity of 88180, by comparison with the attenuated Cu-1 virus.

Complete sequencing of genome segments A and B of IBDV strain 88180 revealed that both genome segments are related to their counterparts in vvIBDV but are nevertheless significantly different. This result confirmed previous analysis performed on partial sequences from this virus strain [40], [44]. Several genetic traits differentiate the 88180 virus from the typical vvIBDV. One of these is a shorter VP5 gene, the longer VP5 gene observed in vvIBDVs (4 more codons in the 5′end of the VP5 ORF as compared with Cu-1 or 88180) being due to the putative start codon being located upstream in vvIBDVs and resulting in a longer VP5 cytoplasmic domain in typical vvIBDVs) [35]. This difference did not appear to be a critical determinant for pathogenicity, as mc88180 proved as pathogenic as strain 89163, our vvIBDV control, in the first experiment. A second major difference between 88180 and vvIBDV affects the VP2 protein. It has been observed that all vvIBDV identified to date share the four vvIBDV-specific VP2 aa positions A222, I256, I294 and S299 [49]. Of these, only I256 is present in 88180. Finally, eleven aa changes scattered along the VP1 protein also differ between 88180 and vvIBDV (Table 1). Three of these differences were located at aa positions 147, 393 and 562, which have otherwise been suggested to be conserved in all vvIBDV [39]. The multiplicity of these differences did not allow to pinpoint any particular region of the genome as responsible for the differences in pathogenicity between 88180 and typical vvIBDVs, which is the reason why in the second part of the study mosaic viruses were constructed instead of correction by site-directed mutagenesis of single mutations.

Reassortant viruses derived from mcCu-1 and mc88180 were constructed in order to check whether some genome segments or the compatibility between the segment A- and segment B- encoded proteins could be major determinants of the 88180 pathogenic phenotype. Although A88BCU1 apparently replicated to the same extent as mc88180, we observed in two independent animal experiments that this virus was significantly less pathogenic than mc88180 (no mortality and a significantly lower morbidity were observed with A88BCU1, the latter observation led to the development of a symptomatic index implemented in the last experiment to quantify IBD signs). This suggests that BCU1 played a role in the reduced pathogenicity of this reassortant virus. This hypothesis is in agreement with a previous study by our group showing that a naturally occurring reassortant virus, with a vvIBDV-derived segment A and an unrelated segment B, had a reduced pathogenicity in spite of a replication ability at 4 DPI similar to that of pathogenic IBDVs [40]. The assumption that segment B may have an influence on pathogenicity even when it does not reduce the replication ability is somewhat in contrast with a previously published study suggesting that segment B of vvIBDV was not involved in pathogenicity [35], and with subsequent work by the same group suggesting that replacement of the segment B of a vvIBDV strain by its counterpart in a cell-culture adapted virus causes attenuation of the resulting reassortant, due to a delayed replication [38]. However, the experimental conditions (different source of SPF chickens and markedly reduced age at challenge and challenge dose in Boot et al., 2005 [38]) could explain these differences.

mc88180 and A88BCU1 but not ACU1B88 induced morbidity, mortality (at least for mc88180), and severe bursal lesions. This suggests that segment A of 88180 played a critical role in determining the pathogenicity of the reverse genetics-generated viruses. This is unlikely to be linked to bursa tropism only, as ACU1 has also been shown previously to confer the ability to replicate in bursal cells [36]. However, ACU1 encodes VP2 aa 253H, 279N and 284T which have been shown in various combinations both to confer the ability to replicate in chicken embryo fibroblasts [50]–[54], and to result in the attenuation of cell-culture-adapted viruses [53], [54]. Our results are hence consistent with the assumption that segment A of 88180 carries the major determinants for pathogenicity, as suggested in vvIBDVs by Boot et al. [35] and van Loon et al. [53]. Whether these determinants lie in those aa that are conserved in vvIBDVs and 88180, such as VP2 aa position I 256 will deserve further study.

In addition to the predominant role of segment A88 in pathogenicity, our results also demonstrated the limited compatibility of segments ACU1 and B88. Indeed, reassortant virus ACU1B88 was not pathogenic (as mentioned above, segment ACU1 most likely played a role in this lack of pathogenicity) and replicated poorly as compared with all other viruses inoculated in the same experiment. This low replication capacity was already apparent at the rescue step. It could not be improved by one further passage of this virus on SPF chickens (data not shown), which explains why the ACU1B88 challenge in experiment ***ii)*** was performed with the original low-titer virus stock. As both the mcCu-1 and mc88180 replicated well in the bursa of the inoculated chickens in the same experiment, the lower replication of ACU1B88 cannot be attributed to a single genome segment, but rather to a low segment compatibility. A lower or delayed replication capacity has also been described in laboratory constructed reassortant IBDV, either derived from the Cu-1 and the serotype 2 23/82 strains [36] or from the variant GLS-TC (tissue culture adapted) and variant GLS-BD (Bursa adapted) strains [37], or between vvIBDV strain D6948 and cell culture adapted CEF94 [35], [38]. Impairment of replication could be due to the disruption of some interactions described to occur between both genome segments and/or the proteins they encode. Indeed, VP1 is known to interact with VP3 [55]–[57], and comparison of the aa sequence of the Cu-1 and 88180 proteins reveals that one out of three and twelve out of 23 aa differences do occur in the interaction domains identified in VP3 and VP1, respectively. Similarly, VP3 interacts with the genome segments [55], [58]–[62], which interaction could be modified by aa changes at polyprotein positions 981 and 990. Finally, VP1 was also described to interact with the 5′ [20], [63] and 3′ NCRs [19], and the NCRs of Cu-1 and 88180 were indeed predicted to have differences in their secondary structures (the 3′ NCRs have been proved to be important for IBDV replication and/or pathogenicity, both by the number of their unpaired terminal cytosines and by their sub terminal hairpin secondary structure [64], [65]). Additional disrupted interactions could affect VP4, which was described to *trans*-activate the synthesis of VP1 [11], however the VP1 and VP4 domains involved in these mechanisms are yet unknown.

Quite discrepantly as compared with ACU1B88, segment incompatibility was not observed with the other reassortant virus, A88BCU1, which induced bursal damage and the same virus titer as mc88180 in BF at 4 DPI (as detected with a quantitative real-time PCR targeted at the genome-specific minus strand of segment A). Hence, a reassortant virus with a segment B derived from a cell-culture-adapted IBDV strain (mcCu-1) replicated well in the bursa. This is in contrast with previous reports studying reassortant viruses derived from bursa- or cell-culture- propagated viruses [37], [38], which reports suggested that the RdRp of cell-culture-adapted or bursa propagated viruses might have a better efficacy for genome replication in cell culture or in the bursa, respectively.

To determine more precisely if specific regions of segment BCU1 could be responsible for the reduced pathogenicity of A88BCU1, we rescued five viruses with mosaic B segments and tested them for their *in vivo* pathogenicity. The exchanged regions within segment B of Cu-1 and 88180 were defined based on the previously determined putative functional motifs within the IBDV RdRp (Figure 1A). Such an approach has not been implemented in Birnaviruses so far, in contrast with another model of RNA viruses, Picornavirus, for which studies based on mosaic polymerases derived from Coxsackie B3 and poliovirus type 1 demonstrated that different domains and several macromolecular interactions are required for efficient delivery of the polymerase to strand-specific replication complexes [66], [67].

Our results with mosaic IBDV's showed that, consistently with the 88180 origin of their segment A, all recombinant viruses were indeed pathogenic as they induced an atrophy of the BF, significant histological lesions and an efficient antibody response. Nevertheless, they all induced significantly less morbidity than mc88180 and none of them caused mortality, although their mosaic RdRps all retained the ability to replicate segment A of 88180 to the same extent, at least as detected with quantitative RT-PCR at 4 DPI. These results show the discrepancy between the virus replication (or fitness) and the pathogenicity of the mosaic viruses. The low fitness and pathogenicity of the reassortant ACU1B88 virus suggested that these two traits are both affected in this virus. However, for A88BCU1 and the mosaic viruses their fitness and virulence seem to be two separate traits. This is somewhat comparable with what has been described for the foot-and-mouth disease virus (FMDV), for which viral fitness and virulence are two different virus traits [68]. Herrera et al showed that a biological clone of FMDV with a high fitness and virulence (cell killing capacity) that undergo plaque to plaque transfers had a profound fitness loss, but with only a minimal decrease of virulence. The authors showed that fitness-decreasing mutations and virulence determinants are distinct and can follow different evolutionary trajectories. For A88BCU1 and the mosaic viruses the absence of link between the virus fitness and virulence remains to be understood.

Our mosaic segment B experiments showed that the D1 and especially the D2 domains play an important role in pathogenicity. The reduced pathogenicity in viruses with heterologous D1 and D2 domains (no mortality and significantly less morbidity compared to mc88180) suggests that the compatibility between D1 and D2 of the RdRp, (Finger 1 of D1 and Palm 2 and Thumb of D2), which is affected when one out these two domains comes from a different genetic background, is required for pathogenicity, although the disruption does not interfere critically with the ability of the viruses to replicate. Interestingly, the recombinant virus incorporating only domain D2 of Cu-1 was milder than its D1 counterpart as it induced significantly less bursal atrophy and histological lesions than B88[D1CU1] or mc88180. This suggests that the Palm 2 and Thumb subdomains of the central domain and C-terminal domain of the polymerase could be involved in functions or interactions more important for pathogenicity. Whether the more important role of D2 could be related to one of the D2 aa differences between Cu-1 and 88180 that is located in motif E of the RdRp is not yet known.

The same level of replication of the mosaic viruses compare to mc88180 suggests that irrespective of their mosaic structure, all RdRps interacted efficiently with the 3′ and 5′ NCRs originating either from Cu-1 or from 88180. Hence, incompatibility of the segment A- and segment B- NCRs was not the only cause for the reduced pathogenicity of A88BCU1. However, the mirror experiment remains to be performed and it cannot be excluded that introducing partial or full length NCRs of Cu-1 in the B88 segment would also result in attenuation. Altogether, these results can hardly compare with a previous study by Schröder et al. [69], [70], showing that incorporating segment A NCRs derived from an apathogenic serotype 2 IBDV strain into the genetic context of a cell culture adapted serotype 1 virus reduced the *in vitro* replication ability but only had a limited influence on bursal lesions. The different impact of the NCR exchange is most probably due to the secondary structures of the exchanged NCRs being very different in the two studies (inter-serotypic exchange in Schröder et al., intra-serotypic exchange in the present work).

Finally, our results also showed that restoring the catalytic domain Dc of 88180 (Palm 1, Finger 2 and beginning of Palm 2 subdomains of the central polymerase domain, containing the C, A and B motifs [21], [22]) in A88BCU1[Dc88], did not result in an increased pathogenicity, even when it was also associated with the 88180 NCRs and either D1 (N-ter region, and Finger 1 region of the central domain [21], [22]) or D2 (Finger 2 and Thumb subdomains of the central domain and C-terminal region [21], [22]). This suggests that VP1 positions 390–391 are not critical for the RdRp activity, a finding which is consistent with these two amino acid changes occurring in the “flexible region” connecting the α1 helice and β1 tube in the Palm 1 region [21], [23], and the A motif where a single mutation was described to completely abolish the RdRp activity [19].

Altogether, our results suggest that restoration of the full pathogenicity of A88BCU1 could require ***i)*** either the incorporation of both the D1 and D2 of B88 (corresponding to the N-terminal region and Finger 1 domain combined with the Palm 2, Thumb subdomain and C-terminal region which included most of the aa differences found between BCU1 and B88180), or ***ii)*** possibly the restoration of the whole interaction domain of the RdRp with VP3 [55], which almost corresponds to the central domain of the polymerase (region overlapping the D1 and D2), or ***iii)*** even the restoration of the whole B88 segment. This demonstrates that different regions of the polymerase are essential for IBDV pathogenicity, even without any impact on virus replication. This is evocative of results reported in Picornaviruses, in which complex interactions involving different domains of the polymerase (that may be remote from the catalytic site) critically contribute to the control of the fidelity of the polymerase, [71], which in turn determines the genetic heterogeneity between the quasi species in the viral population and may so modify the organ tropism and pathogenicity of mutant viruses, although they replicate to the same extent [72]. However, the mechanisms of the reduction of pathogenicity in the recombinant IBDVs still deserves further study. It cannot be ruled out that changes in the early replicating cycle of the virus, before 4 days post inoculation, or changes in the host immune responses at the early stages of infection could be involved.

## Materials and Methods

### Ethics statement

All experiments were performed in agreement with national regulations on animal welfare and animal experiments, according to authorisations N°22-4 and B-22-745-1, by Prefecture des Cotes-d'Armor, France. The rescue of IBDV recombinant viruses and their in vivo characterization (see below) were performed according to authorization n° 3569 CA-I by the French Commission for Genetically Modified Organisms.

### Viruses and titration

IBDV strain 88180 [44], which is not adapted to cell culture, was used as the parental strain for the construction of the molecularly cloned 88180 virus (mc88180). The 89163 [47] and Faragher 52/70 [73] IBDV strains were used as the references for vvIBDV and classical IBDV strains, respectively. Virus suspensions, produced from the bursa of inoculated chickens, were used as inocula [47]. Inocula were titrated by inoculating serial ten-fold dilutions (0.1 ml per egg, chorio allantoic membrane route, seven eggs per dilution) to 9-to-10-day-old SPF eggs (Anses-Ploufragan/Plouzané laboratory). IBDV titers were calculated according to the method of Reed & Muench (1938) [74] and expressed as median embryo infectious doses (EID_50_).

### Cells and chickens

Transfection experiments were performed using CEF derived from SPF hen eggs (Anses-Ploufragan/Plouzané laboratory) grown in BHK-21 medium (Eurobio) supplemented with 0.3% Tryptose Phosphate Broth (TPB), 5% foetal calf serum (FSC), 200 U/ml antibiotics (streptomycin and penicillin) and 2 µg/ml fungizone. Animal experiments were performed using five-to-seven-week-old SPF White Leghorn chickens (Anses-Ploufragan/Plouzané laboratory) maintained in level 3 containment facilities in filtered-air negative-pressure isolation units.

### Sequencing of the full-length A and B segments of IBDV strain 88180

Virus RNA was extracted from infected bursae as described previously [75]. The coding regions of segments A and B were sequenced using DNA fragments resulting from overlapping reverse transcription (RT)- polymerase chain reactions (PCR) as previously described [40], (oligonucleotide primers available upon request). The 5′ and 3′ non coding regions (NCR) of segments A and B were sequenced using the 5′ Rapid Amplification of cDNAs Ends (5′RACE; Invitrogen) according the manufacturer's protocol, using IBDV specific primers (the sequences of the oligonucleotides used in this study are presented in supplementary Table S2). For tailing, either dGTP or dCTP were used in independent reactions, to determine the first or last nt of each extremity. The tailed cDNA was then used for two consecutive PCR with nested virus-specific primers and poly(C)- or poly(G)-primers (Table S2). PCR products were purified as described above and were digested with SpeI and EcoRI before ligation into the similarly cleaved pMOSBLUE vector (Amersham Pharmacia Biotech). Transformation into competent One shot top10 *E.coli* (Invitrogen) of the resulting plasmids followed by plasmid extraction (Qiagen) were performed according to the manufacturer's protocol. Sequencing of the resulting cDNA clones was repeated until at least five clones with the longest and same nucleotide sequence had been analysed.

### Construction of full-length cDNA clones of the A and B segments of 88180

Full-length single stranded cDNA copies of the A and B segments of the 88180 strain were produced by RT as described above, using oligonucleotide primers complementary to the 3′ end of the non-coding strand of segments A and B ( = “sense” primers) (Table S2). The two sense primers contained 5′ extensions carrying the T7 promoter preceded by either the *EcoRI* or the *XbaI* restriction site in primers designed for segments A or B, respectively. The resulting cDNA was amplified in PCR reactions performed as described above and based on primer pairs consisting of the same sense primer as used for RT, combined with an antisense primer with a 5′ extension corresponding to a cloning site (*EcoRI* or *SmaI* for segments A or B, respectively) followed by a linearisation site (*BsRGI* for segment A, for segment B the *SmaI* site used for cloning was also used as a linearisation site). The conditions for the amplification of the full-length segments were 35 cycles with denaturation at 95°C for 30 sec, annealing for 45 sec (at 63.8°C or 61.7°C for segments A or B, respectively) and extension at 72°C for 3 min. The PCR products were purified as above. After enzymatic digestion of the PCR products with *EcoRI* only (segment A) or *XbaI* and *SmaI* (segment B), each segment was ligated to the similarly cleaved pUC18 vector. The resulting plasmids containing the segment A or B sequence, designated as pA88 and pB88 for segments A and B, respectively, were transformed into *E. coli*, purified and sequenced on both strands as described above. Comparison of the full-length A and B segment sequences derived from the bursa propagated 88180 virus (used as the reference sequences) with the corresponding sequences of full-length cloned segment A and B (pA88 and pB88, respectively) was performed. Three unintended nt mutations (nts 972, 3261 and 3262) and one (nt 2828) were present in pA88 and pB88, respectively. Mutation at nt 972 of pA88 was non-silent and induced the mutation from a Glycine residue to a Glutamic acid at position 281 and was conserved as a genetic tag to differentiate the molecular clone mc88180 to 88180 virus. The three other mutations, nts 3261, 3262 in pA88 and nt 2828 in pB88, were missing cytosine nts in the 3′ extremity of the 3′ NCRs and were corrected by site directed mutagenesis (Stratagene) with specific primers as described in Table S2.

### Construction of mosaic B segments by combining different regions of the 88180 and Cu-1 genomes

Segment B of strain Cu-1 was obtained as pUCCu-1B, a plasmid with a similar construction as pB88 [36]. To ensure consistency in the names of the recombinant viruses, pUCCu-1B was renamed pBCU1 in the present study. The putative functional motifs and different domains of IBDV VP1 are shown in Figure 1 A and B respectively, together with the number and positions of aa changes (coding regions) or nt changes (NCR) which differentiate IBDV strains 88180 and Cu-1 (Figure 1 C). Based on these data, the *SspI*, *BstXI*, *PmLI* and *BsRGI* conserved and unique restriction sites (position shown at Figure 1 C) were selected to construct five new plasmids derived from pB88 and pBCU1 (Figure 1 D and E). The new constructs corresponded to the introduction into a pBCU1 background of 88180 ***i)*** NCRs, ***ii)*** catalytic domain (Dc) , ***iii)*** both the NCRs and the Dc ***iv)*** the NCRs, the Dc and the 5′ coding part of VP1 (D1) and ***v)*** the NCRs, the Dc and the 3′ coding part of VP1 (D2) (Figure 1 E).

***i)*** To construct a clone of segment B of Cu-1 with the NCRs of 88180, the *SspI*-*BsRGI* fragment (approximately 3.2 kbp) was first excised from pB88 and replaced by its counterpart similarly derived from pBCU1, to obtain pBCU1[NCR3′ 88]. Then, the *Ssp1*-*BstXI* fragment of pB88 containing the 5′NCR of 88180 was subcloned into the similarly cleaved pBCU1[NCR3′ 88] to obtain pBCU1[NCRs88], corresponding of the B segment of Cu-1 with both the 5′ and 3′ NCRs of 88180. ***ii)*** and ***iii)*** The two mutations typical of Dc of 88180 (Figure 1 C) were introduced into the pBCU1 and pBCU1[NCRs88] plasmids by site-directed mutagenesis using specific primers (Table S2), resulting in plasmids pBCU1[Dc88] and pBCU1[NCRsDc88], respectively. ***iv)*** To construct a clone of segment B of 88180 with D2 of Cu-1, the *BstXI*-*PmLI* fragment of pB88 was subcloned into the similarly cleaved pBCU1[NCRsDC88] to obtain pB88[D2CU1]. ***v)*** Finally, to construct a full- length clone of segment B of 88180 with D1 of Cu-1, the *PmLI*-*BsRGI* fragment of pB88 was subcloned into the similarly cleaved pBCU1[NCRsDC88] to obtain pB88[D1CU1]. The sequences of the cloned or mutated regions in pBCU1[NCRs88], pBCU1[Dc88] and pBCU1[NCRsDC88] were checked and the sequences of the two last constructs, plasmids pB88[D2CU1] and pB88[D1CU1], were completely determined as described above.

### Construction of a transcription/transfection plasmid control

Two primers (sequence available upon request) complementary to the 3′ extremities of both the coding and non-coding strands of the Green Florescent Protein gene (GFP, accession number U57609) were used to amplify the GFP gene from the pcDNA3.1/Zeo/GFP plasmid, in a PCR as described above. The sense primer contained a 5′ extension carrying the T7 promoter preceded by a *PstI* restriction site whereas the antisense primer carried an *XmaI* site. After *PstI*-*XmaI* digestion, the PCR product was cloned into the similarly cleaved POP24+(100A) containing a polyA [76] to obtain the POP24+GFP100A plasmid with the GFP gene under the control of the T7 promotor and a polyA at the 3′ extremity followed by an *EcoRI* restriction site. In order to use the pUC18 plasmid (vector also used for the construction of full-length cDNA clones of the A- and B- segments of 88180 and Cu-1 strains) the *PstI*-*EcoRI* fragment of POP24+GFP100A was subsequently ligated into the similarly cleaved pUC18 plasmid to obtain the plasmid pT7GFPpolyA with the GFP gene under the control of the T7 promotor and with the *EcoRI* site used as linearisation site. Upon *in vitro* transcription this plasmid generates a 1047 nt- long GFP cRNA ending with a stretch of 100 A nucleotides, thus mimicking a polyadenylated GFP mRNA.

### *In vitro* transcription

The plasmids containing the full-length cDNA copies of the native or chimeric IBDV genome segments or the GFP gene were linearised with the restriction enzymes specific for the restriction sites introduced at their 3′ ends by the primers listed in Table S2. The linearised plasmids were then used as templates for *in vitro* transcription as described by Mundt and Vakharia (1996) [45] with minor modifications as described by Zierenberg *et al.* (2004) [36]. The *in vitro* transcription reaction was incubated for 1 h at 37°C. Synthesis of cRNA was confirmed by electrophoresis of 2 µl of the reaction mixture in a 1% agarose gel and visualisation under UV light.

### Rescue of infectious IBDV

The capped cRNAs were used, in various combinations, for transfection of CEF, as described previously [36], [45]. Efficacy of *in vitro* transcription/transfection was checked using pT7GFPpoly100A by visualizing fluorescence under UV light in the transfected cells at 48 h post transfection. Forty eight hours after transfection, the cells were scraped and harvested with supernatants and inoculated by the intramuscular route to SPF chickens (1 ml per bird, five birds per virus). Four days after inoculation, the chickens were humanely killed and their bursae were harvested and processed for virus isolation as described previously [47]. The genetic make up and the references of the rescued viruses are presented in Table S1. The genetic organization of the rescued IBDV was checked for conformity with the expected construction by nucleotide sequencing as described above.

### Comparison of the antigenic profile of 88180 and mc88180

The parent virus and its molecular clone were antigenically characterized using a previously described antigen capture ELISA (AC-ELISA) based on a panel of 8 different neutralizing anti-IBDV Mabs detecting at least 6 epitopes of the VP2 protein [44], [77].

### Animal experiments

Three experiments were performed to compare the pathogenicity of ***i)*** 88180 and mc88180, ***ii)*** mc88180, mcCu-1 and the corresponding reassortant viruses (A88 BCU1 and ACU1 B88) and ***iii)*** mc88180 and the recombinant viruses derived from mosaic B segments. A summary of each experimental design is presented in supplementary Table S3. On the first day of the experiments, SPF chickens were housed in groups of comparable sex and weight and each chicken in each group was blood sampled to check for seronegativity versus IBDV. On the same day, each bird was inoculated by the intranasal route with a standardized dose of the relevant virus (10^5^ EID_50_ by bird except in experiment ***ii***) where the ACU1 B88 virus was inoculated with 10^3.6^ EID_50_ by bird). In each experiment a group was kept as a mock-inoculated control receiving PBS only. Clinical signs until 10 days post inoculation (DPI) and cumulated mortality rates were measured. Bursae and blood were sampled at different times post infections. At each sampling date (Table S3), at least five birds per group were weighed, humanely killed, necropsied and their bursae were weighed for the subsequent calculation of the b/B ratio. At 4 DPI, each bursa was cut in two halves after weighing, one half was kept for histological examinations, the other was processed for the preparation of a virus suspension (see below). All surviving birds at the end of the experiments (Table S3) were humanely killed and sampled for final serological and histological examinations of the BF. The criteria analysed to assess the virus acute pathogenicity were the intensity of the clinical signs, the mortality rate, the IBDV-induced bursal atrophy (as measured by the b/B ratio), the severity of the bursal histological lesions (see below) at 4 DPI and at the end of experiment, the serological responses and the virus titers in bursae harvested at 4 DPI (Table S3).

### Symptomatic index

The intensity of the clinical signs was more precisely evaluated in the last animal experiment by two different experimenters, using individual marking of the birds (with coloured rings on legs) and a symptomatic index that we specifically designed for the quotation of IBD symptoms. This index ranges from 0 to 3, with 0 meaning “lack of signs”, 1 meaning “typical IBD signs (ruffled feathers) conspicuous in quiet bird only, the bird stimulated by a sudden change in environment (light, noise, or vicinity of experiment observer) appears normal, motility is not reduced”; 2 meaning “typical IBD signs conspicuous even when bird is stimulated, dehydration is apparent, motility may be slightly reduced” and 3 standing for “typical severe IBD signs with prostration or death”. In the third experiment, the mean symptomatic index (MSI) of the surviving chickens was calculated daily from days 1 to 10 PI for each inoculated virus.

### Histological examinations

All histological examinations were performed by Dr M. Lagadic (Maisons-Alfort, France). Bursae of Fabricius from the different groups and from the different sampling times were examined individually. The severity of the IBD-induced histological lesions was quantified according to Skeeles' scale for bursal lesion scores (BLS) [78] which is based on five degrees: 0 = no lesions; 1 = mild scattered cell depletion in a few follicles; 2 = moderate, one-third to one half of the follicles have atrophy or depletion of cells; 3 = diffuse, atrophy of all follicles; and 4 = acute inflammation and acute necrosis typical of IBD.

### IBDV neutralization test

Virus neutralization (VN) tests were performed in CEF, using 100 median tissue infective doses (TCID_50_) per well of the CT IBDV strain (serotype 1), as described previously [77]. The VN titer was expressed as log_2_ of the last dilution resulting in 100% neutralization of the cytopathogenic effect. VN titers higher than 3.3 were considered as positive.

### Quantification of virus RNA using quantitative RT-PCR

To easily quantify the virus in the virus suspension prepared from the individual BF harvested at 4 DPI, we designed a Taqman©-based quantitative RT-PCR (qRT-PCR) for IBDV segment A. A pair of suitable primers and a FAM-labelled TAMRA-quenched probe (Table S2) were defined using the Primer Express software (version 2.0, Applied Biosystems), in a region of segment A (nts 3031–3092) that proved to be sufficiently conserved in the alignment of pA88 and pACU1. The latter plasmid corresponds to the cloned full-length segment A of Cu-1 and was obtained as pUCCu-1A [36]. For consistency grounds pUCCu-1A was renamed pACU1 within the present study. The qRT-PCR assay was designed with discrete RT and PCR steps and targeted at the RT step the minus strand of the virus RNA (present in mature IBDV particles only).

Briefly, a sample of BF (approximately 250 mg) was first ground weight/vol in PBS. The supernatant harvested after centrifugation at 12500 rpm for 10 min at +4°C was used for virus RNA extraction (QIAmp viral RNA mini kit, Qiagen). The “high capacity cDNA archive kit” (Applied Biosystems) was used for RT. The RT mix (25 µl of denatured RNA, 1× of RT buffer, 1× of a dNTP mixture, 400 nM of the sense primer, 125 units of Multiscribe RT and RNase-free water to a final volume of 50 µl) was incubated at 37°C for 1 h. The PCR was then performed according to the manufacturer's protocol in a 96-well format using the ABI Prism® 7000 Sequence Detection System (Applied Biosystems). The reaction mixture (2.5 µl of the RT product, 1× of TaqMan Universal Master mix, 200 nM of probe, 150 nM of each primer and RNase-free water to a final volume of 25 µl) was subjected to the following thermocycling conditions: 50°C, 2 min; 95°C, 10 min; 40 cycles of 95°C, 15 sec, 60°C 1 min. All cDNA samples were tested in duplicates. Data were analysed using the Sequence detection version 1.2.3 software (Applied Biosystems). The final results were expressed as cycle threshold (Ct) value, which is the cycle when the change in the emission of fluorescence by the reporter dye significantly passes a background threshold calculated by the software. Ct values were converted into virus titers (EID_50_) using a standard curve prepared from serial 10 fold dilutions of aliquoted and repeatedly titrated reference suspensions of the F52/70, 89163, mc88180 or mcCu-1 viruses.

### Statistical analysis

Percent values (morbidity and mortality rates) were compared using the Chi squared test. Small series of quantitative values were compared using the non parametric Kruskal-Wallis test for the one-way analysis of variance. All statistical analysis were performed using version 9.0 of the Systat software (Systat Software, Inc.).

### Submission of nucleotide sequences to databases

The full-length nt sequences for segments A and B of IBDV strain 88180 were submitted to the EMBL database with accession numbers AM111353 and AM111354, respectively.

## Supporting Information

Table S1 **References of the viruses rescued from the different combinations of cloned segments A and B.** pACU1 and pBCU1 correspond to plasmids pUCCu-1A and pUCCu-1B, respectively, described by Zierenberg et al. (2004) [36].
(DOC)

Table S2 **Sequence of the oligonucleotide primers and probe used in this study.**
(DOC)

Table S3 **Summarized protocol of the three animal experiments.** The three animal experiments were designed to: *i)* compare the pathogenicity of 88180 with mc88180, *ii)* compare the pathogenicity of mc88180, mcCu-1 and reassortant viruses derived thereof and *iii)* compare the pathogenicity of mosaic segment-B derived viruses.
(DOC)
