# Supplementary material for: Different Domains of the RNA Polymerase of Infectious Bursal Disease Virus Contribute to Virulence
Source: PLoS One. 2012 Jan 13;7(1):e28064. doi: 10.1371/journal.pone.0028064 (PMC3258228; doi:10.1371/journal.pone.0028064)
Supplement: Table S1 — References of the viruses rescued from the different combinations of cloned segments A and B. pACU1 and pBCU1 correspond to plasmids pUCCu-1A and pUCCu-1B, respectively, described by Zierenberg et al. (2004) [36]. (DOC) [file pone.0028064.s001.doc]

**Table S1 :** **References of the viruses rescued from the different combinations of cloned segments A and B.**

| Plasmid used forSegment A | **Plasmid used for**  **segment B** | **Reference of**  **rescued virus** |
| --- | --- | --- |
| pA88 | pB88 | mc88180 |
| pACU1 | pBCU1 | mcCU1 |
| pA88 | pBCU1 | A88 BCU1 |
| pACU1 | pB88 | ACU1 B88 |
| pA88 | pBCU1[NCRs88] | BCU1[NCRs88] |
| pA88 | pBCU1[Dc88] | BCU1[Dc88] |
| pA88 | pBCU1[NCRsDc88] | BCU1[NCRsDc88] |
| pA88 | pB88[D1CU1] | B88[D1CU1] |
| pA88 | pB88[D2CU1] | B88[D2CU1] |

pACU1 and pBCU1 correspond to plasmids pUCCu-1A and pUCCu-1B, respectively, described by Zierenberg et al. (2004) [36].
