# Supplementary material for: Different Domains of the RNA Polymerase of Infectious Bursal Disease Virus Contribute to Virulence
Source: PLoS One. 2012 Jan 13;7(1):e28064. doi: 10.1371/journal.pone.0028064 (PMC3258228; doi:10.1371/journal.pone.0028064)
Supplement: Table S2 — Sequence of the oligonucleotide primers and probe used in this study. (DOC) [file pone.0028064.s002.doc]

**TABLE S2: Sequence of the oligonucleotide primers and probe used in this study**

| Primer | Nucleotide sequence | Purpose | Polarity | Restriction sites and position |
| --- | --- | --- | --- | --- |
| -702 | ACATGTGGCTACCATTTTTG | 5’RACE RT NCR5’ segtA | Antisense | 702-721 |
| -341 | TTGCTCTGCAGTGTGTAGTG | 5’RACE PCR1 NCR5’ segtA | Antisense | 341-360 |
| -197 | CG**GAATTC**ATGGACGCCGGTCCGGT | 5’RACE PCR2 NCR5’ segtA | Antisense | **EcoRI**; 197-213 |
| +2229 | GCCTTAAGTTGGCTGGTCCC | 5’RACE RT NCR3’ segtA | Sense | 2229-2248 |
| +2609 | AAGTCGCAAAGGGCCAAGTA | 5’RACE PCR1 NCR3’ segtA | Sense | 2609-2628 |
| +2906 | CG**GAATTC**ACGTCGATCTACGGGGCTCCAG | 5’RACE PCR2 NCR3’ segtA | Sense | **EcoRI**; 2906-2927 |
| -1229 | TAAACGGGTTGAACTTGT | 5’RACE RT NCR5’ segtB | Antisense | 1229-1246 |
| -466 | GTACGCATTGGGCTTTTC | 5’RACE PCR1 NCR5’ segtB | Antisense | 466-483 |
| -246 | **GAATTC**GGCCAGACGACTAGGGCTG | 5’RACE PCR2 NCR5’ segtB | Antisense | **EcoRI**; 246-264 |
| +1985 | CGTTGAGGTTGGTAGGTG | 5’RACE RT NCR3’ segtB | Sense | 1985-2002 |
| +2399 | ATGACCCCGATGCAGACT | 5’RACE PCR1 NCR3’ segtB | Sense | 2399-2416 |
| +2522 | **GAATTC**TCCAGTCAACCTCAGTGT | 5’RACE PCR2 NCR3’ segtB | Sense | **EcoRI**; 2522-2539 |
| AAP(C) | GGCCACGCGTCG**ACTAGT**ACCCCCCCCCCCCCCCCC | 5’RACE PCR1 |  | **SpeI**; |
| AAP(G) | GGCCACGCGTCG**ACTAGT**ACGGGGGGGGGGGGGGG | 5’RACE PCR1 |  | **SpeI**; |
| AUAP | GGCCACGCGTCG**ACTAGT**AC | 5’RACE PCR2 |  | **SpeI**; |
| HN13 | ACCG**GAATTC***TAATACGACTCACTATA*GGATACGATCGGTCTGA | RT full length segment A | Sense | **EcoRI**; 1-17 |
| HN13 | ACCG**GAATTC***TAATACGACTCACTATA*GGATACGATCGGTCTGA | PCR full length segment A | Sense | **EcoRI**; 1-17 |
| HN14 | ACGCGGTACCGACAG**GAATTC**GGCTT**TGTACA**GGGGACCCGCGAACGGATCCAATT | PCR full length segment A | Antisense | **EcoRI BsrGI**; 3237-3260 |
| HN15 | CTAG**TCTAGA***TAATACGACTCACTATA*GGATACGATGGGTCTGAC | RT full length segment B | Sense | **XbaI**; 1-18 |
| HN15 | CTAG**TCTAGA***TAATACGACTCACTATA*GGATACGATGGGTCTGAC | PCR full length segment B | Sense | **XbaI**; 1-18 |
| HN16 | ACCGCTCGAGTCTAGA**CCCGGG**GGCCCCCGCAGGCGAAG | PCR full length segment B | Antisense | **SmaI**; 2808-2827 |
| A3’(1) | CGTTCGCGGGTCCCCCCTGTACAAAGCC | Correction of cloned A segment | Sense | Nts added; *3246-3260* |
| A3’(2) | GGCTTTGTACAGGGGGGACCCGCGAACG | Correction of cloned A segment | Antisense | Nts added*; 3246-3260* |
| B3’(1) | CTGCGGGGGCCCCCCGGGTACCGAGCTCG | Correction of cloned B segment | Sense | Nts added; *2814-2827* |
| B3’(2) | CGAGCTCGGTACCCGGGGGGCCCCCGCAG | Correction of cloned B segment | Antisense | Nts added; *2814-2827* |
| BCU1(Dc)(1) | GGATCGTCGAGTGGATAATGACCCCGGAAGAACCCAAG | Mutation of the catalytic Domain | Sense | Nts mutated*;* *1262-1299* |
| BCU1(Dc)(2) | CTTGGGTTCTTCCGGGGTCATTATCCACTCGACGATCC | Mutation of the catalytic Domain | Antisense | *Nts mutated*; 1262-1299 |
| Gu SegA 3031 | GACTGCGATGGAGATGAAGCA | Quantitative RT-PCR primer | Sense | 3031-3051 |
| Gu SegA 3073 | GTTTTGGCTTGGGCTTTGGT | Quantitative RT-PCR primer | Antisense | 3073-3092 |
| Gu SegA 3053 | CGCAATCCCAGGCGGGCTC | Quantitative RT-PCR probe | Sense | 3053-3071 |

The primers are listed 5’ to 3’. Bold face indicates the restriction sites introduced into the chimeric primers to allow for the easy cloning of the RT-PCR products. Italic and underlined faces indicate the T7 promotor sequence and the mutated nucleotides, respectively. Nucleotide positions within IBDV segments are indicated by reference to the full-length genome sequence of IBDV strain 88180 (Acc ***AM111353*** and ***AM111354*** for segment A and B, respectively).
