# Supplementary material for: Different Domains of the RNA Polymerase of Infectious Bursal Disease Virus Contribute to Virulence
Source: PLoS One. 2012 Jan 13;7(1):e28064. doi: 10.1371/journal.pone.0028064 (PMC3258228; doi:10.1371/journal.pone.0028064)
Supplement: Table S3 — Summarized protocol of the three animal experiments. The three animal experiments were designed to: i) compare the pathogenicity of 88180 with mc88180, ii) compare the pathogenicity of mc88180, mcCu-1 and reassortant viruses derived thereof and iii) compare the pathogenicity of mosaic segment-B derived viruses. (DOC) [file pone.0028064.s003.doc]

**TABLE S3:** **Summarized protocol of the three animal experiments.**

| **Animal experiments** | **Virus inoculated** | **Number of groups**  **(chicken by group)** | **Age of chickens**  **(in weeks)** | **Sampling dates** | **Method of virus quantification in BF** |
| --- | --- | --- | --- | --- | --- |
| ***i)*** | F52/70  89163  88180  mc88180  mock | 5 (30) | 6 | 4, 17 | qRT-PCR |
|  |  |  |  |  |  |
| ***ii)*** | mc88180  mcCU1  A88 BCU1  ACU1 B88  mock | 5 (20) | 7 | 4, 20 | qRT-PCR |
|  |  |  |  |  |  |
| ***iii)*** | mc88180  A88 BCU1  BCU1[NCRs88]  BCU1[Dc88]  BCU1[NCRsDc88]  B88[D1CU1]  B88[D2CU1]  mock | 8 (20) | 5 | 4, 21 | qRT-PCR |

The three animal experiments were designed to: *i)* compare the pathogenicity of 88180 with mc88180, *ii)* compare the pathogenicity of mc88180, mcCu-1 and reassortant viruses derived thereof and *iii)* compare the pathogenicity of mosaic segment-B derived viruses.
